# HIV patients treated with low-dose prednisolone exhibit lower immune activation than untreated patients

**DOI:** 10.1186/1471-2334-12-14

**Published:** 2012-01-20

**Authors:** Christa Kasang, Albrecht Ulmer, Norbert Donhauser, Barbara Schmidt, August Stich, Hartwig Klinker, Samuel Kalluvya, Eleni Koutsilieri, Axel Rethwilm, Carsten Scheller

**Affiliations:** 1 University of Wuerzburg, Institute of Virology und Immunobiology, 97078 Wuerzburg, Germany; 2 HIV-Intensive Care Unit, Schwabstr. 26, 70197 Stuttgart, Germany; 3 Institute of Virology, Clinical and Molecular Virology, National Reference Center for Retroviruses, University of Erlangen-Nuernberg, Erlangen, Germany; 4 Medical Mission Institute, Department of Tropical Medicine, 97067 Wuerzburg, Germany; 5 University of Wuerzburg, Medical Clinic and Policlinic II, Josef-Schneider-Str. 2, 97080 Wuerzburg, Germany; 6 Bugando Medical Center, Mwanza, Tanzania

## Abstract

**Background:**

HIV-associated general immune activation is a strong predictor for HIV disease progression, suggesting that chronic immune activation may drive HIV pathogenesis. Consequently, immunomodulating agents may decelerate HIV disease progression.

**Methods:**

In an observational study, we determined immune activation in HIV patients receiving low-dose (5 mg/day) prednisolone with or without highly-active antiretroviral therapy (HAART) compared to patients without prednisolone treatment. Lymphocyte activation was determined by flow cytometry detecting expression of CD38 on CD8(+) T cells. The monocyte activation markers sCD14 and LPS binding protein (LBP) as well as inflammation markers soluble urokinase plasminogen activated receptor (suPAR) and sCD40L were determined from plasma by ELISA.

**Results:**

CD38-expression on CD8+ T lymphocytes was significantly lower in prednisolone-treated patients compared to untreated patients (median 55.40% [percentile range 48.76-67.70] versus 73.34% [65.21-78.92], *p *= 0.0011, Mann-Whitney test). Similarly, we detected lower levels of sCD14 (3.6 μg/ml [2.78-5.12] vs. 6.11 μg/ml [4.58-7.70]; *p *= 0.0048), LBP (2.18 ng/ml [1.59-2.87] vs. 3.45 ng/ml [1.84-5.03]; *p *= 0.0386), suPAR antigen (2.17 μg/ml [1.65-2.81] vs. 2.56 μg/ml [2.24-4.26]; *p *= 0.0351) and a trend towards lower levels of sCD40L (2.70 pg/ml [1.90-4.00] vs. 3.60 pg/ml [2.95-5.30]; *p *= 0.0782). Viral load in both groups was similar (0.8 × 10^5 ^ng/ml [0.2-42.4 × 10^5^] vs. 1.1 × 10^5 ^[0.5-12.2 × 10^5^]; *p *= 0.3806). No effects attributable to prednisolone were observed when patients receiving HAART in combination with prednisolone were compared to patients who received HAART alone.

**Conclusions:**

Patients treated with low-dose prednisolone display significantly lower general immune activation than untreated patients. Further longitudinal studies are required to assess whether treatment with low-dose prednisolone translates into differences in HIV disease progression.

## Background

Progressive depletion of helper T cells is a hallmark of untreated HIV infection. There is accumulating evidence that chronic immune activation may be a fundamental driving force of this T cell loss. HIV infection triggers a general activation of the immune system that persists for years, which may eventually result in exhaustion of the regenerative capacities of the immune system, causing immunodeficiency and AIDS [1-3]. The trigger for this chronic stimulation is probably multifactorial, including a direct stimulation of HIV-specific cells by the ongoing replication of HIV [4]. Furthermore, elevated levels of plasma lipopolysaccharide (LPS) and bacterial DNA in chronic HIV patients suggest that microbial translocation from the damaged gut contributes to this hyperactivation by triggering an innate immune response to Gram-negative bacteria [5,6]. During this process, the two host factors LPS binding protein (LBP) and soluble CD14 neutralize plasma LPS and direct it to the Toll-like receptor (TLR)-4 molecule expressed on macrophages [7,8].

The pivotal role of chronic immune activation in HIV pathogenesis is underlined by the fact that immune activation parameters such as expression of CD38 or HLA-DR on CD8+ T are good predictors of subsequent T cell loss and correlate much better with HIV disease progression than plasma viral load or chemokine coreceptor usage [2]. In a similar way, plasma levels of soluble sCD14, LBP and soluble urokinase plasminogen activator receptor (suPAR) correlate with HIV disease progression [2,9,10]. The transmembrane glycoprotein CD40L (CD154), a member of the tumor necrosis factor family, is primarily expressed on activated CD4+ T cells, but also detected on many other cell populations including a small proportion of CD8+ cells [11]. The soluble form (sCD40L) still binds to the receptor and delivers biological signals in a cytokine-like manner [12]. Increased sCD40L levels were detected in the serum of HIV-infected patients [13].

Chronic immune activation as a consequence of immunodeficiency virus infection is restricted to man and Asian monkeys, who both develop AIDS following untreated HIV/SIV infection. In contrast, African monkeys which do not exhibit chronic immune activation following SIV infection do not progress to AIDS and remain healthy, despite high viral load [6,14,15].

Treatment with antiretroviral therapy does not only suppress virus replication but also substantially reduces HIV-associated general immune activation [16-21]. Compared to uninfected controls, HAART-treated patients however still exhibit slightly elevated immune activation that is being considered a risk factor for ongoing disease progression (albeit at much slower pace than in untreated patients) [22-27]. Despite the dramatic success of antiretroviral therapy on reduction of mortality in HIV infection, HIV-infected patients treated with HAART still have a life expectancy below the average of the uninfected population [28]. Immune-based therapies that aim to further reduce immune activation under HAART may therefore further close this gap. In resource-limited areas with later onset of HAART compared to more industrialized countries, cheap and robust immunomodulating regimens preceding antiretroviral therapy may be another option to reduce worldwide HIV mortality until HAART becomes universally available.

In this study we investigated general immune activation in HIV patients who received low-dose (5 mg/day) prednisolone in combination with or without HAART compared to untreated patients.

## Methods

### Subjects

The patients analyzed in this study received HIV-treatment according to the treatment recommendations of the "Deutsche AIDS Gesellschaft" (DAIG). In addition to antiretroviral medication, some of our patients also received low-dose (5 mg/day) prednisolone as part of their individual treatment plan. Among the 101 HIV patients included into our study, 27 received low-dose (5 mg/day) prednisolone, 31 received low-dose prednisolone in combination with HAART, 30 received HAART alone and 13 received neither HAART nor prednisolone.

### Prednisolone medication

We (A. Ulmer) have previously reported that HIV patients who do not yet meet the eligibility criteria for antiretroviral therapy seem to profit from treatment with low-dose (5 mg per day) prednisolone by a stabilization of helper T cell counts compared to patients that remained therapy-naive [29,30]. Due to these encouraging observations, patients at the site of A. Ulmer are being offered low-dose prednisolone (prior or accompanying to HAART) and patients decide individually of whether or not to receive prednisolone. We therefore supervise a population of patients that can be categorized into four subgroups: untreated-patients, prednisolone-treated patients, and at later stages of the infection HAART-treated patients who receive or do not receive prednisolone. All patients investigated in this study derive from the same study center (A. Ulmer), which was the only participating site that could enroll prednisolone-treated patients. To compare immune activation in patients with therapeutic (HAART) and immunologic control of virus replication, we included 3 additional treatment-naïve patients (elite controllers who suppress virus replication beyond the detection limit of 50 copies/ml) from another site (H. Klinker).

### Study design

In an observational study we compared immune activation parameters in 5 different subject groups: (1) HIV-1-infected subjects who receive neither HAART nor prednisolone and with detectable viral load, referred to as "untreated"; (2) HIV-1 infected subjects treated with 5 mg/day prednisolone, referred to as "Prednisolone"; (3) HIV-1 infected subjects treated with antiretroviral therapy, referred to as "HAART"; (4) HIV-infected subjects treated with antiretroviral therapy in combination with 5 mg/day prednisolone, referred to as "HAART+Prednisolone"; (5) HIV-1 infected subjects as described for (1) but with undetectable viral load referred to as "elite controllers". Patients have been asked to participate in the study during their routine visits at the site. Patients willing to participate in the study signed an informed consent. No additional selection criteria other than willingness to participate were applied for inclusion into the study.

The study was approved by the ethical committees of the Landesärztekammer Baden-Württemberg, the Bayerische Landesärztekammer, and the University of Würzburg.

### Isolation of plasma

7.5 ml of whole blood were collected from each patient with a vacutainer supplemented with EDTA (BD Biosciences) for flow cytometric analysis of PBMC. EDTA plasma was used for sCD40L-ELISA. Additional 8 ml of blood were collected for plasma preparation from heparin-blood. Heparin-plasma was used for sCD14- and LBP-ELISAs.

### Flow cytometry

Fresh EDTA-blood was used within 8 h after collection. Erythrocytes were lysed using a lysis solution (BD Biosciences) and PBMC were stained in triplicates with antibodies directed at CD3 (labeled with FITC) and CD8+ (labeled with PerCP) and counterstained with anti-CD38-PE (all antibodies from were purchased from BD Biosciences, Heidelberg, Germany) according to the "lysis no wash" protocol (BD Biosciences). Cells were analyzed by flow cytometry using a FACS-Calibur flow cytometer (Becton Dickinson). Markers were set according to cells stained with fluorochrome-conjugated isotype control antibodies (all from BD Biosciences).

Lymphocytes were identified by gating in a dot plot of forward and sideward scatter (FSC/SSC). For analysis of T cell activation, cells were stained with anti-CD3(PerCP)/CD8(FITC)/CD38(PE)-antibodies. CD3+/CD8+ cells within the FSC/SSC lymphocyte gate were scored as CD8+ T-lymphocytes. CD3+/CD8- cells within the FSC/SSC lymphocyte gate were scored as CD4-positive T cells. CD38 expression was determined in CD3+/CD8+ lymphocytes (CD8 T lymphocytes) and CD3+/CD8- lymphocytes (scored as CD4+ T lymphocytes). CD38+ lymphocytes were scored as activated cells. For analysis of naïve T cells, cells were stained with anti-CD3(PerCP)/CD4(APC)/CD45RA(FITC)/CD62L(PE)-antibodies. CD3+/CD4+ cells within the FSC/SSC lymphocyte gate were scored as CD4+ T-lymphocytes. CD3+/CD4- cells within the FSC/SSC lymphocyte gate were scored as CD8-positive T cells. CD45RA/CD62L-expression was determined in CD3+/CD4+ lymphocytes (CD4 T lymphocytes) and CD3+/CD4- lymphocytes (scored as CD8+ T cells). CD45RA/CD62L-positive lymphocytes were scored as naïve cells.

### Viral load and CD4 counts

CD4 counts and viral load were determined from EDTA-blood by a commercial virusdiagnostic laboratory during routine medical care (CD4: Prof. Enders, Stuttgart, viral load: Dr. Jaegel-Guedes/Jäger, München).

### sCD14, LBP, suPAR and sCD40L

ELISAs to detect plasma levels of sCD14 (Diaclone), LBP (Hycult Biotech), suPAR (ViroGates) and sCD40L (Bender MedSystems) were performed according to the manufacturer's instructions. For the latter, 100 μl of EDTA plasma samples were analyzed that had been thawed once.

### Statistical analysis

Statistical analysis was performed using the GraphPad Prism software (version 4.0c for Macintosh). About half of the data showed a non-Gaussian distribution (according to D'Agostino-Pearson test) so that medians and interquartile range (expressed as 25%-75% percentile) were chosen to describe the results. Differences between untreated and Prednisolone-treated or HAART-treated and HAART + Prednisolone-treated groups were analyzed by Mann-Whitney *U*-test and *p *< 0.05 was regarded as statistically significant. Correlations were performed using linear regression.

## Results

### Patient population

In this observational study we analyzed general immune activation parameters in patients with HIV infection receiving individual therapy regimes. Patients who were willing to participate in the study gave written informed consent and donated a single blood sample. We divided the patients into five different groups, including a) untreated patients (n = 10), b) patients being treated with low-dose prednisolone (n = 27), c) with HAART (n = 30), d) HAART plus prednisolone (N = 31) or e) treatment-naïve patients with undetectable viral load (Table 1). At the time of blood sampling, the 27 patients in the prednisolone group received prednisolone medication for a median duration of 2.3 years (Table 1). Although the exact time of infection for each patient is unknown, the date of the first HIV diagnosis indicates that patients in the prednisolone and HAART groups are probably much longer infected with HIV (median of 9 years in both HAART groups and median of 5 years in the prednisolone-only group) than patients in the no-treatment group (median of 2 years) (Table 1).

**Table 1:** Patient characteristics

Treatment group	Untreated (n = 10)	Prednisolone (n = 27)	HAART	Prednisolone/HAART	no treatment, elite controllers
number of patients	10	27	30	31	3

age in years	39 [28.0-50.5]	38 [32.0-47.0]	47 [42.5-61.0]	48 [40.0-58.0]	28, 43, 56*

sex m/f	9 m/1 f	22 m/5 f	24 m/6 f	23 m/8 f	2 m/1 f

time since first HIV diagnosis in years	2 [1.0-4.0]	5 [3.0-13.0]	9 [4.5-16.0]	9 [3.0-14.0]	3, 15, 21*

prednisolone treatment in years	n.a.	2.3 [1.2-3.5]	n.a.	5 [2.0-5.7]**	n.a.

viral load (RNA copies/ml plasma)	1.1 × 10^5 ^[0.5-12.2 × 10^5^]	0.8 × 10^5 ^[0.2-42.4 × 10^5^]	27 undetectable, others [[46;66;191]]	25 undetectable, others: [[41; 46; 63; 74; 131; 1085]]	undetectable

CD4+ counts (cells/μl)	520 [350-670]	550 [460-700]	465[320-655]	390[280-600]	284, 554, 892*

Values represent medians [interquartile range as 25%-75% percentile]. *) As the number of elite controllers in this study is only 3, all three single values are depicted instead of the median. Cutoff for viral load (VL) for all patients is 40 copies/ml plasma, except for the three patients (elite controllers) derived from H. Klinker with a detection limit of 50 copies/ml. Values in double brackets [[x]] represent VL of individual HAART-treated patients with detectable VL. **) For 7 patients treated with HAART+prednisolone the exact time of prednisolone treatment was unknown but their medical records documented treatment for more than 5 years. For the calculation of the median, the time of treatment for these patients was set to 5 years

### Viral load

Patients in the no-treatment and prednisolone-treatment groups presented with comparable amounts of viral load (medians 1.1 × 10^5 ^and 0.8 × 10^5^, respectively) (Table 1). Most of the patients who received HAART (both in combination with or absence of prednisolone) had undetectable viral load (below 40-50 copies/ml).

### CD38-expression on T cells

**Figure 1:**
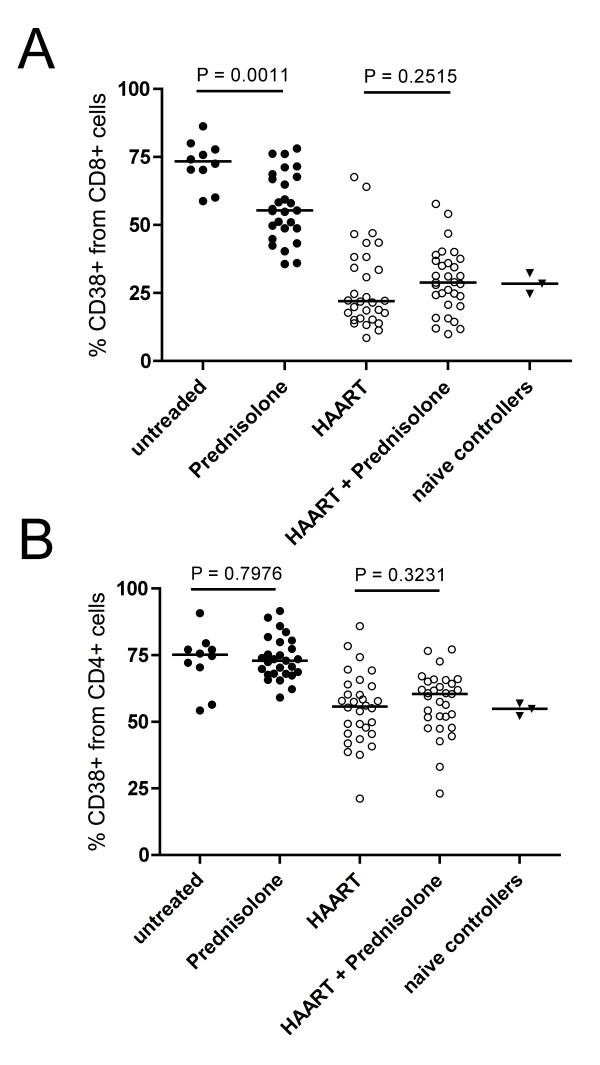
**Prednisolone medication in untreated HIV infection is associated with lower CD8+ T cell activation**. Whole blood from donors of different treatment groups was stained with fluorescent dye-coupled anti-CD3, anti-CD8 and anti-CD38 antibodies. Cells were analyzed by flow cytometry after erythrocyte lysis. Lymphocytes were gated in a FSC/SSC scatter. CD3+/CD8+ cells from the lymphocyte gate were scored as CD8+ T cells, CD3+/CD8- cells were scored as CD4+ T cells. Horizontal bars represents medians. Statistical analysis was performed using a Mann-Whitney *U *test. **A**: Percentage of CD8+ T cells expressing CD38. **B**: Percentage of CD4+ T cells expressing CD38

T cell activation was studied by flow cytometric analysis of whole blood stained with anti-CD3/CD8/CD38. CD3-positive/CD8-negative cells were scored as CD4-positive T lymphocytes. Patients in the prednisolone group exhibited statistically significant lower activation of CD8+ T cells than patients in the untreated group patients (median 55.40% [percentile range 48.76-67.70] versus 73.34% [65.21-78.92], *p *= 0.0011). (Figure 1A). The lowest activation rates were measured in HAART and HAART+prednisolone-treated patients (median 22.07% [15.49-38.27] versus 28.85% [20.69-36.87]) and prednisolone comedication had no detectable effect on CD8-activation in HAART-treated patients (*p *= 0.2515). CD38-expression on CD8 T cells in patients receiving HAART was similar to CD38 expression in "elite controllers" but due to the very limited number of patients in this group (n = 3) we will not go into further details.

Prednisolone medication had apparently no effect on CD38 expression in CD8-negative T cells, independent of whether patients received prednisolone in the absence or presence of HAART (Figure 1B)

### sCD14 and LBP

**Figure 2:**
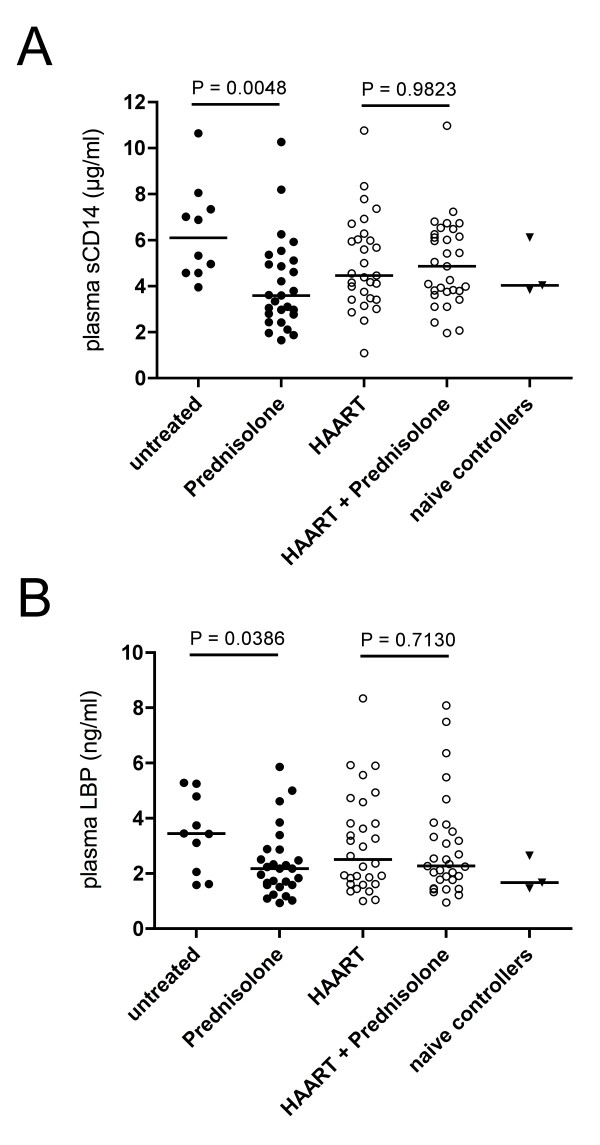
**Prednisolone medication in untreated HIV infection is associated with lower monocyte activation**. sCD14 and LBP concentrations were determined by ELISA from plasma from donors of different treatment groups. Horizontal bars represents medians. Statistical analysis was performed using a Mann-Whitney *U *test. **A**: Plasma concentrations of sCD14. **B**: Plasma concentrations of LBP.

In order to determine the effects of low-dose prednisolone on monocyte activation associated with the LPS-response, we analyzed levels of soluble CD14 (sCD14) and LPS-binding protein (LBP). We found significantly lower concentrations of plasma sCD14 in patients treated with prednisolone alone compared to untreated patients (3.6 μg/ml [2.78-5.12] vs. 6.11 μg/ml [4.58-7.70]; *p *= 0.0048) (Figure 2A). No effects attributable to prednisolone could be observed in HAART-treated patients. Similar to what we found for sCD14 levels, patients in the prednisolone-only group showed significantly lower LBP-levels compared to untreated patie2Ants (2.18 ng/ml [1.59-2.87] vs. 3.45 ng/ml [1.84-5.03]; *p *= 0.0386) (Figure 2B), and again no additional effects related to prednisolone were observed in HAART-treated patients.

### suPAR and sCD40L

**Figure 3:**
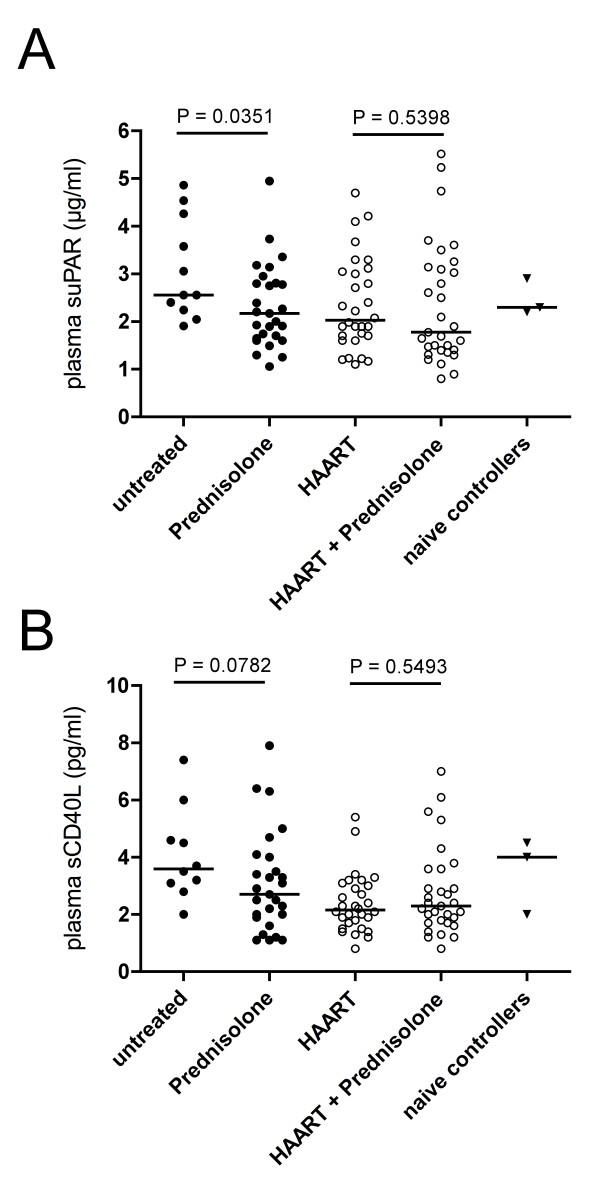
**Prednisolone medication in untreated HIV infection is associated with lower inflammation**. Concentrations of suPAR and sCD40L were determined by ELISA from plasma from donors of different treatment groups. Horizontal bars represents medians. Statistical analysis was performed using a Mann-Whitney test *U*-test. **A**: suPAR. **B**: sCD40L.

Comparing patients treated with prednisolone alone and untreated patients, significantly lower concentrations of plasma suPAR levels were detected in prednisolone-treated patients (2.17 μg/ml [1.65-2.81] vs. 2.56 μg/ml [2.24-4.26]; *p *= 0.0351) as well as a strong trend towards lower levels of sCD40L (2.70 pg/ml [1.90-4.00] vs. 3.60 pg/ml [2.95-5.30]; *p *= 0.0782) (Figure 3B). In the presence of HAART, no effects attributable to prednisolone could be observed for the two markers (Figure 3A, B).

### CD4 T cell counts, CD4/CD8 ratio and naïve cells

**Figure 4:**
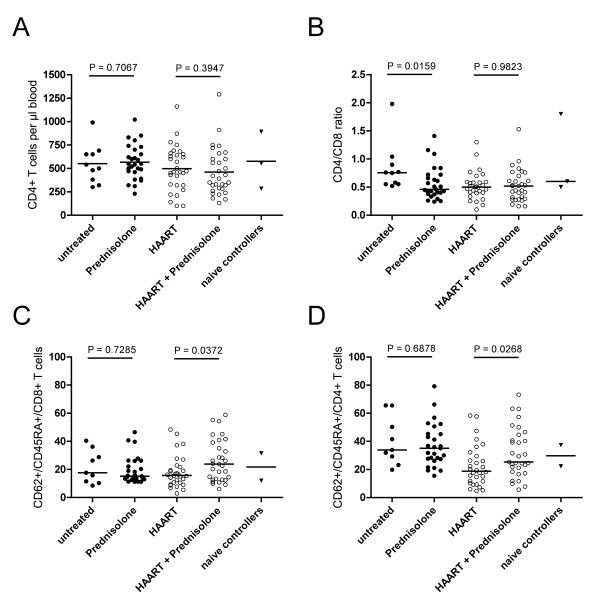
**The influence of prednisolone on CD4 counts, CD4/CD8 ratio and naïve T cells**. CD4 counts and CD4/CD8 ratio were determined from blood samples by routine diagnostic. Whole blood from donors of different treatment groups was stained with fluorescent dye-coupled anti-CD3, anti-CD4, anti-CD62L and anti-CD45RA antibodies. Cells were analyzed by flow cytometry after erythrocyte lysis. Lymphocytes were gated in a FSC/SSC scatter. CD3+/CD4+/CD62L+/CD45RA cells from the lymphocyte gate were scored as naïve CD4+ T cells, CD3+/CD4-/CD62L+/CD45RA cells were scored as naïve CD8+ T cells. Horizontal bars represent medians. Statistical analysis was performed using a Mann-Whitney test. **A**: absolute CD4+ T cell counts. **B**: CD4/CD8 T cell ratio. **C**: percentage of naïve CD8+ T lymphocytes. **D**: percentage of naïve CD4+ T lymphocytes.

As depicted in Figure 4A, patients treated with prednisolone-only presented with CD4+ T cell counts comparable to untreated patients (550 cells/μl [460-700] vs. 520 cells/μl [350-670]; *p *= 0.7067). Patients treated with HAART or HAART/prednisolone exhibited a trend towards lower CD4 T cell counts compared to therapy naïve and prednisolone-treated patients. The CD4/CD8 ratio in prednisolone-only-treated patients was significantly decreased compared to treatment-naïve patients (ratio 0.46 [0.38-0.72] vs. ratio 0.755 [0.55-0.97]), and comparable to HAART and HAART/prednisolone-treated patients (Figure 4B). As the CD4+ T cell counts in prednisolone-treated and naive patients are similar (Figure 4 A), the decrease of the observed CD4/CD8 ratio in prednisolone-treated patients reflects a relative increase of the CD8+ T cell population. This increase could be a direct consequence of the observed reduced immune activation of this population (Figure 1A), which may translate into reduced turnover rates.

In patients treated with HAART, prednisolone-medication was associated with a significantly higher percentage of memory (CD62L+/CD45RA+) CD8+ T cells (23.8% [12.6 - 39.1] vs. 15.8% [9.84-22.25]; *p *= 0.0372) (Figure 4C) and memory CD4+ T cells (25.4% [18.3 - 44.6] vs. 18.8% [10.9-30.85]; *p *= 0.0268) (Figure 4D). No effects attributable to prednisolone on the frequency of naive T cells were detected between untreated and prednisolone-only-treated patients.

## Discussion

In this study we investigated factors of general immune activation associated with HIV disease progression, and we found significantly lower values in patients receiving prednisolone compared to untreated patients, suggesting that prednisolone may have beneficial effects on immunological correlates of HIV disease progression in otherwise untreated patients. In patients treated with HAART, no additional beneficial effects attributable to prednisolone could be observed. The immune-modulating activity of prednisolone in otherwise untreated patients described in this manuscript is in line with previous observations, reported by Andrieu et al., who observed in an uncontrolled, open-label study an increase in CD4+ T cell counts following administration of 0.3-0.5 mg/kg prednisolone over a period of 12 months [31]. Prednisolone treatment (with a concentration about 4-8 times higher than in our study) was accompanied by a reduction of immune activation. Patients were encouraged to continue 0.3 mg/kg prednisolone as follow up-medication as long as their CD4+ T cell levels remained above baseline levels. Follow-up prednisolone treatment postponed CD4+ T cell decrease (relative to baseline levels) by two years [32]. A few patients experienced relatively mild prednisolone-associated side effects such as face swelling, an increase in body weight and elevated blood pressure.

In another, randomized, placebo-controlled trial, prednisolone was given for 8 weeks with a decreasing dose from 50 mg to 15 mg per day; the main outcome was survival [33]. Elliot and colleagues observed only a trend towards better survival in prednisolone-treated patients compared to HIV controls (21 versus 25 deaths per 100 person years; difference statistically not significant) but a statistically significant increase in the incidence of Kaposi sarcoma (4.2 versus 0 cases per 100 person years). The study population was not being stratified for HHV-8 seropositivity, the causative factor of Kaposi sarcoma, and HHV-8 infection status was not being analyzed. (In another short-term randomized, placebo-controlled trial studying the effects of prednisolone on effusive tuberculous pericaditis in HIV-infected individuals, with a similar dosage and time scale [60 mg starting dose, tapered with 10 mg per week until completion at the sixths week], three cases of Kaposi sarcoma were observed among the 58 study participants and all of them received placebo [34].) The main difference in the treatment regime towards other studies, including ours, is the relative short duration of prednisolone treatment of only 8 weeks, which may hamper the observation of a therapeutic benefit with the primary endpoint of survival.

Two other randomized, placebo-controlled trials investigated the effects of prednisolone on HIV-infection in the presence of HAART. In a study with a dose of 0.5 mg per kg body weight per day given for 8 weeks, a decrease in CD8+/CD38+ cells was reported with no changes in viral load and CD4+ cell counts [35]. In partial contrast to this, a fixed daily dose of 40 mg prednisolone was given for 8 weeks in another trial, resulting in an increase of CD4+ counts compared to baseline values of 40%, but no changes in HLADR/CD38-positive CD8 cells and naive T cells (CD45RA+/CD62L+) were reported [36].

We have previously analyzed the CD4+ cell counts of some of our patients in a longitudinal observational study and found that long-term treatment with low-dose prednisolone is safe and - similar to what has been found by Andrieu et al. [31] - associated with a postponement of CD4+ T cell loss by about 2 years relative to baseline values [29,30]. Our present study may offer a mechanistic explanation for the T cell preserving activity of low-dose prednisolone, i.e. by reducing general immune activation. Unfortunately, the previously described CD4+ T cell preserving activity of prednisolone cannot be directly addressed in this study (although a trend towards higher counts in prednisolone-treated patients was visible), as this would require a longitudinal analysis and probably also a more homogenous patient sample with respect to time to infection and therapy initiation.

The strong association between general immune activation and HIV disease progression is also seen in studies investigating the effects of HAART on immune activation. Similar to what we found in our study, HAART does not only suppress virus replication but also reduces immune activation [18-20]. In some patients - the so-called virological nonresponders - HAART fails to suppress virus replication but effectively restores immune activation and CD4 T cell counts [37]. On the other hand, immunological nonresponders, who do not regenerate helper T cell counts despite HAART-mediated suppression of virus replication, show enhanced immune activation [38]. Massanella et al. reported in a cross sectional study that patients with limited CD4 T-cell repopulation under HAART exhibited high T-cell activation rates (CD38+, HLA-DR+), suggesting that immunomodulating strategies should be envisaged to treat discordant patients [39].

General immune activation not only correlates with progression of immunodeficiency, but also with neurological complications of HIV infection. HIV patients with cognitive impairment or brain atrophy exhibit more elevated sCD14 levels than neurologically asymptomatic HIV-positive controls [40]. This link between monocyte activation and the neurological complications of HIV infection fits well into the overall picture in which microglia activation has been identified as a correlate with immunodeficiency virus-mediated neuropathy [41,42].

Due to the non-prospective and non-randomized design of our study, our findings should be interpreted with caution, as potential yet undiscovered confounding mechanisms may have biased the result. Moreover, the low sample size of our study is relatively low and differences in individual viral loads (although not statistically significant between untreated and prednisolone-treated patients) are possible limitations. As we found significantly lower immune activation in patients treated with prednisolone compared to untreated patients, the critical reader might object that this difference might have been caused by an unintended enrichment of natural slow-progressors in the group of prednisolone-treated patients over time, who would lower the average immune activation in this group due to intrinsically lower immune activation in these patients. (The argument in this case would be that patients who progressed under prednisolone sooner or later would have left this group to become treated with HAART. Hence, over time, patients with an intrinsic slower disease progression would have become enriched in this group. At a closer look, this mechanism would, however, also apply for patients in the untreated group - compared to which prednisolone-treated patients exhibited significantly lower activation.) We therefore checked whether there is any correlation in prednisolone-treated patients between immune activation (CD8/CD38, sCD14, LBP, suPAR) and the duration of prednisolone medication or the (known) time of infection and found no correlation whatsoever for any of the markers (P values of correlations ranged between 0.2748 and 0.9399, data not shown). These findings suggest that a prednisolone-independent enrichment over time of slow-progressing patients in this group has not taken place, otherwise one should have expected to observe lower levels of immune activation in patients with longer treatment times.

As for the absence of detectable effects of prednisolone in HAART-treated patients in almost all analyzed parameters in this study, this may well be attributed to the heterogeneity of the patients (differences in CD4 counts, time to infection), differences in the composition of individual antiretroviral regimens that may have different effects on immune activation or to the low dose of prednisolone used. Further studies specifically addressing the effects of prednisolone in the presence of HAART looking at more homogenous patient groups are therefore needed. Studying the effects of immune modulators in the presence of HAART (in contrast to immune modulators preceding HAART) is of particular interest, since there is residual immune activation in HAART-treated patients associated with accelerated disease progression [25].

In countries with lesser access to (and therefore later onset of) HAART, prednisolone medication preceding antiretroviral treatment might be considered as a supplement regimen in HIV therapy, provided that prednisolone-mediated immune modulation is safe and translates into a clinical benefit for the patients. In order to address this question, we are currently performing a randomized, double-blinded, placebo-controlled clinical trial to assess the effects of low-dose prednisolone on immune activation and HIV disease progression in otherwise treatment-naïve patients, recruiting 326 patients in Tanzania (trial name: "ProCort1"; registry: http://ClinicalTrials.gov; registration number: NCT01299948). Recruitment is now completed and first results will be available soon.

## Conclusions

General immune activation is considered to be a key factor for HIV disease progression and the results of our study indicate that treatment with low-dose prednisolone may reduce this activation. Further studies are required to analyze whether attenuation of immune activation achieved by immunomodulatory substances translates into clinical benefit for the patients.

## List of abbreviations

AIDS: acquired immunodeficiency syndrome; EDTA: ethylene-diamin tetraacetic acid; ELISA: enzyme-linked immunosorbent assay; FCS/SSC: forward scatter/sideward scatter; HAART: highly-active antiretroviral therapy; HHV-8: human herpesvirus 8; HIV: human immunodeficiency virus; LBP: LPS-binding protein; LPS: lipopolysaccharide; PBMC: peripheral blood mononuclear cells; SIV: simian immunodeficiency virus; suPAR: soluble urokinase plasminogen activator receptor

## Competing interests

The authors declare that they have no competing interests.

## Authors' contributions

CK, AU, AS, SK and CS participated in the design and coordination of the study. CK, ND, BS, and CS carried out the immune activation studies. BS and CS performed the statistical analysis. CK, AU, BS, AS, HK, EK, AR and CS drafted the manuscript and CS wrote its final version. All authors read and approved the final manuscript.

## Pre-publication history

The pre-publication history for this paper can be accessed here:

http://www.biomedcentral.com/1471-2334/12/14/prepub
